# Knockout of the 15 kDa Selenoprotein Protects against Chemically-Induced Aberrant Crypt Formation in Mice

**DOI:** 10.1371/journal.pone.0050574

**Published:** 2012-12-04

**Authors:** Petra A. Tsuji, Bradley A. Carlson, Salvador Naranjo-Suarez, Min-Hyuk Yoo, Xue-Ming Xu, Dmitri E. Fomenko, Vadim N. Gladyshev, Dolph L. Hatfield, Cindy D. Davis

**Affiliations:** 1 Department of Biological Sciences, Towson University, Towson, Maryland, United States of America; 2 Molecular Biology of Selenium Section, Laboratory of Cancer Prevention, National Institutes of Health, Bethesda, Maryland, United States of America; 3 Office of Dietary Supplements, National Institutes of Health, Bethesda, Maryland, United States of America; 4 Department of Biochemistry, University of Nebraska, Lincoln, Nebraska, United States of America; 5 Brigham and Women’s Hospital, Harvard Medical School, Boston, Massachusetts, United States of America; University of Chicago, United States of America

## Abstract

Evidence suggests that selenium has cancer preventive properties that are largely mediated through selenoproteins. Our previous observations demonstrated that targeted down-regulation of the 15 kDa selenoprotein (Sep15) in murine colon cancer cells resulted in the reversal of the cancer phenotype. The present study investigated the effect of Sep15 knockout in mice using a chemically-induced colon cancer model. Homozygous Sep15 knockout mice, and wild type littermate controls were given four weekly subcutaneous injections of azoxymethane (10 mg/kg). Sep15 knockout mice developed significantly (p<0.001) fewer aberrant crypt foci than controls demonstrating that loss of Sep15 protects against aberrant crypt foci formation. Dietary selenium above adequate levels did not significantly affect aberrant crypt foci formation in Sep15 knockout mice. To investigate molecular targets affected by loss of Sep15, gene expression patterns in colonic mucosal cells of knockout and wild type mice were examined using microarray analysis. Subsequent analyses verified that guanylate binding protein-1 (GBP-1) mRNA and protein expression were strongly upregulated in Sep15 knockout mice. GBP-1, which is expressed in response to interferon-γ, is considered to be an activation marker during inflammatory diseases, and up-regulation of *GBP-1* in humans has been associated with a highly significant, increased five-year survival rate in colorectal cancer patients. In agreement with these studies, we observed a higher level of interferon-γ in plasma of Sep15 knockout mice. Overall, our results demonstrate for the first time, that Sep15 knockout mice are protected against chemically-induced aberrant crypt foci formation and that Sep15 appears to have oncogenic properties in colon carcinogenesis *in vivo.*

## Introduction

Colorectal cancer remains the second leading cause of cancer-related deaths in the United States with an estimated 51,690 deaths (26,470 in men and 25,220 in women) during 2012 [1]. Aberrant colonic crypts and aberrant crypt foci are putative pre-neoplastic colon lesions, and are considered good biomarkers for determining colorectal cancer risk, as the number of pre-neoplastic lesions is statistically associated with the number of tumors that ultimately develop [2], [3]. There is evidence from epidemiologic, clinical and preclinical studies that dietary supplementation with the essential trace mineral selenium reduces the incidence and mortality of colon cancer in humans [4], [5]. Previous animal studies have demonstrated protective effects of selenium fortification against aberrant crypt formation and colon tumor development [5]–[8]. Recent studies indicate that both low molecular weight selenocompounds and selenium-containing proteins (selenoproteins) can mediate the cancer-protective effects of selenium in the colon [9].

Selenium is incorporated into selenoproteins as the 21^st^ amino acid selenocysteine [10], and there are 24 known selenoprotein genes in mice [11]. One of the more abundant selenoproteins in mammals is the 15 kDa selenoprotein (Sep15) [12]. The product of the Sep15 gene belongs to the class of thiol oxidoreductase selenoproteins and is characterized by the thioredoxin-like fold [13], [14]. Sep15 has been suggested to take part in the process of quality control of oxidative protein folding either through rearrangement of disulfide bonds (isomerase function) or reduction of incorrectly formed disulfide bonds (reductase function) in misfolded glycoproteins bound to UDP-glucose:glycoprotein glucosyltransferase (UGGT) [13], [15]. The functional role of Sep15 in cancer remains unclear. In humans, Sep15 is located on chromosome 1p31 [12], a locus commonly mutated in human cancer [16], and decreased expression of Sep15 has been observed in liver, prostate and lung cancer [14]. Previous observations have suggested that a lower expression of Sep15 may be important in promoting carcinogenesis in liver [14] and breast tissue [16], and in approximately 60% of human malignant mesothelioma cell lines and tumors [17]. Most recently, a single nucleotide polymorphism in the Sep15 gene with carriage of a variant allele has also been associated with an increased risk of rectal cancer in Korean men [18].

Our previously published study demonstrated that targeted down-regulation of Sep15 using shRNA (>90% reduction of Sep15 mRNA and protein levels) reversed many of the characteristics typical of cancer cells, including inhibiting anchorage-dependent and anchorage-independent cell growth as well as tumor growth and lung metastasis in mouse colon carcinoma CT-26 but not in mouse Lewis lung carcinoma LLC-1 cells. Targeted down-regulation of Sep15 also inhibited growth of HCT-116 and HT-29 human colon cancer cells [19]. Thus, Sep15 appears to have a tissue-specific role in colon cancer [20].

The purpose of this study was to assess the role of Sep15 knockout on colon cancer risk *in vivo*. Specifically, utilizing a Sep15 knockout mouse model [21], we sought to determine the effects of Sep15 removal on chemically-induced aberrant crypt foci as a measure of pre-neoplastic colon lesions.

## Materials and Methods

### Materials

NuPage® 4–12% polyacrylamide gels, LDS sample buffer, See-Blue Plus2 protein markers, polyvinylidene difluoride membranes and TRIzol® reagent were purchased from Invitrogen (Carlsbad, CA) and 5,5′-dithio-bis(2-nitrobenzoic acid) and aurothioglucose (ATG) from Sigma-Aldrich (St. Louis, MO). Antibodies against Sep15, selenoprotein M (SelM) and thioredoxin reductase 1 (TR1) were generated in our laboratories using recombinant Sep15, SelM or TR1 as antigens. Rabbit polyclonal glutathione peroxidase 1 (GPx1) antibody was purchased from Abcam (Cambridge, MA). Monoclonal antibody against β-catenin was obtained from Cell Signaling Technology, Inc. (Danvers, MA). Rabbit anti-GPx2 antibodies were generated by Dr. Paul Goldsmith, National Cancer Institute, National Institutes of Health (Bethesda, MD) and validated in our laboratory.

Goat polyclonal actin and guanylate binding protein-1 (GBP-1) primary antibodies, and horseradish peroxidase-conjugated secondary antibody were obtained from Santa Cruz Biotechnology (Santa Cruz, CA), SuperSignal West Dura substrate from Pierce (Rockford, IL). iScript™ cDNA synthesis Kit and SYBR® green supermix were purchased from Bio-Rad Laboratories (Philadelphia, PA). Primers for real-time RT-PCR were purchased from Sigma-Genosys (St. Louis, MO). A mouse TH1/TH2 9-Plex assay kit was purchased from MesoScale Discovery (Gaithersburg, MD). Other reagents used were commercially available and were of the highest available quality.

### Animal Studies

#### Ethics statement

Mice were handled and humanely sacrificed in strict accordance with the National Institutes of Health Institutional Guidelines under the expert direction of Dr. John Dennis (NCI, NIH, Bethesda, MD, USA) and all mouse experiments were approved by the Animal Ethics Committee at the National Institutes of Health.

Sep15 knockout mice lacking exon 2 of the gene, and thus lack the functional Sep15 protein, have been generated as described [21]. Mice were bred in-house and maintained in a temperature- and humidity-controlled animal facility with a 12-h light/dark cycle. Animals were given free access to de-ionized water, and were monitored closely for any clinical signs of poor health throughout the study. Male weanling homozygous Sep15 knockout mice (Sep15^−/−^), heterozygous (Sep15^+/−^) and wild type (Sep15^+/+^) littermate controls were maintained on a selenium-deficient Torula yeast-based diet that was supplemented with 0 µg, 0.1 µg or 2.0 µg selenium/g diet as sodium selenite (Teklad, Harlan Laboratories, Madison, WI). Genotypes of the animals were verified by PCR using the following primers:

Wild type allele detection (250 bp): 5′-CAGAGTTTGCGTCAGAGGCATGCAGAG-3′ and 5′-CTGAAACTCGTAAAGTCAGAGACTACTGG-3′; knockout allele detection (312 bp): 5′-GGTGTGTTTGCAGATAAGCTAATGC-3′ and 5′-TACCCGGTAGAATTGACCTGCAG-3′.

### Aberrant Crypt Foci Analysis

Male Sep15 knockout mice (Sep15^−/−^), heterozygous (Sep15^+/−^) and wild type (Sep15^+/+^) littermate controls (N = 12/genotype) were maintained on selenium-adequate diet (0.1 µg selenium/g diet) for three weeks, then given four weekly subcutaneous injections of azoxymethane (AOM, 10 mg/kg). In a parallel study, Sep15 knockout mice (N = 8–11/diet) were maintained on selenium-deficient, adequate, or supranutritionally supplemented diets (0, 0.1 or 2.0 µg selenium/g diet, respectively) for three weeks before injections with AOM and for the remainder of the study. Eight weeks after the last treatment, all animals were sacrificed, and tissues harvested. Livers were excised, snap frozen and stored at −80°C for future analyses. Colons were excised from anus to caecum, rinsed with PBS, opened longitudinally, fixed and stored in 70% ethanol for a minimum of 48 hours. Colons were stained with methylene blue (1 g/L in PBS, pH 7.4), and aberrant crypts and foci were counted using a dissecting microscope by a trained scientist who was blinded to genotype and dietary treatment.

### Tissue Collection

Sep15^−/−^, Sep15^+/−^ and Sep15^+/+^ littermate controls (N = 10/genotype) were maintained on selenium-deficient, adequate, or supranutritionally supplemented diets (0, 0.1 or 2.0 µg selenium/g diet, respectively) for six weeks, sacrificed, and tissues were harvested. Livers were excised, snap frozen and stored at −80°C for future analyses. Blood was collected by cardiac puncture, centrifuged in heparinized tubes at 6,000 rpm for 5 min, and plasma was snap frozen and stored at −80°C for further analysis of cytokines. Colons were excised from anus to caecum, rinsed with cold PBS, opened and colonic epithelia were scraped for further analysis of mRNA and protein expression.

### Real time RT-PCR Analysis

Total RNA was extracted from colonic epithelia using TRIzol® Reagent. cDNA was synthesized using an iScript™ cDNA synthesis kit with 1 µg of total RNA. For real-time RT-PCR, 1.5 µl of cDNA was used in 20 µl reactions using the DNA Engine Opticon® 2 Real-Time RT-PCR Detection System (MJ-Research/BioRad Laboratories, Hercules, CA). The primers used for real-time RT-PCR are shown in Table S1. The mRNA levels of selenoproteins were calculated relative to the expression of glyceraldehyde-3-phosphate dehydrogenase (GAPDH), which was utilized as the internal control.

### Western Blot Analysis

Colonic epithelia were scraped and homogenized in lysis buffer. Extracted proteins were electrophoresed at 40–100 µg/lane on NuPAGE® 4–12% polyacrylamide gels followed by transferring to polyvinylidene difluoride membranes. The membranes were blocked in 5% non-fat dry milk in Tris-buffered saline with 0.1% Tween 20 (TBST) for a minimum of 1 hour and incubated with primary antibodies against murine Sep15, TR1, SelM, GPx1, β-catenin, or GBP-1 overnight (1∶500 in 5% non-fat dry milk/TBST). Horseradish peroxidase-conjugated secondary antibody (1∶10,000) was applied for 1–2 hours, and the membranes were incubated in chemiluminescent substrate and exposed to X-ray film. For detection of GBP-1, the more sensitive Odyssey® Infrared Imaging System was employed, for which the membrane was incubated for one hour in the dark with a LI-COR IRDye®680 secondary antibody (1∶4,000). The membrane was then washed with TBST, dried for at least 2 hours and then exposed to the LI-COR Odyssey Infrared Imager (model 9120, LI-COR Biosciences, Lincoln, NE). For both types of Western analysis methods, β-actin was used as an internal loading control.

### Microarray Analysis

mRNA was isolated from colonic epithelia of wild type (Sep15^+/+^) and Sep15 knockout (Sep15^−/−^) mice maintained on an adequate selenium diet (0.1 µg Selenium/g diet; N = 4/genotype). Microarray analysis was performed on Affymetrix Mouse 430 2.0 gene chips containing 45,000 gene probes. Four arrays were analyzed per genotype, each containing an mRNA sample from an individual mouse. Results per genotype were grouped and compared by ANOVA and those genes significantly different from the wild type mice (p<0.001) were subjected to Ingenuity Pathway Analysis (IPA v. 7.5, Redwood City, CA), which significantly linked genes according to the biological processes in which they function.

### Cytokine Analyses

Mouse blood was collected by cardiac puncture from mice (N = 5/genotype), centrifuged for 5 min (6,000 rpm) in heparanized tubes, and plasma was snap-frozen and stored at −80°C. Using the mouse TH1/TH2 9-Plex assay kit, protein levels of interferon-γ, interleukin-1β, interleukin-2, interleukin-4, interleukin-5, KC/GRO, interleukin-10, interleukin-12p70 and tumor necrosis factor-α were assessed in a sandwich immunoassay format using a SECTOR® Imager 2400 according to the manufacturer’s instructions (MesoScale Discovery, Gaithersburg, MD). An eight-point standard curve was used to calculate the concentration of analytes in the plasma samples. Samples and standards were assayed in duplicates.

### Statistical Analyses

Data are presented as means ± SEM. Data were analyzed by ANOVA followed by Tukey’s Multiple Comparison *post-hoc* test using GraphPad Prism (v.4; La Jolla, CA). The level of significance was set at p<0.05.

## Results

### Animal Weights and Aberrant Crypt Foci Analysis

Male Sep15 knockout (Sep15^−/−^), heterozygote (Sep15^+/−^) and wild type (Sep15^+/+^) mice were maintained on a diet containing adequate selenium levels at 0.1 µg selenium/g diet, and were injected subcutaneously with azoxymethane (AOM). Tissues were collected eight weeks after the final dose, and colons were inspected for aberrant crypt foci as putative pre-neoplastic colon lesions. Animals were monitored closely throughout the study. No significant differences were observed in animal weight at the end of the study or weight gain over the course of AOM treatment among Sep15^−/−^, Sep15^+/−^ or Sep15^+/+^ mice. The final weights in grams were 27.0±0.85, 28.1±0.79 and 29.0±0.54 (mean±SEM, N = 12), respectively.

**Figure 1:**
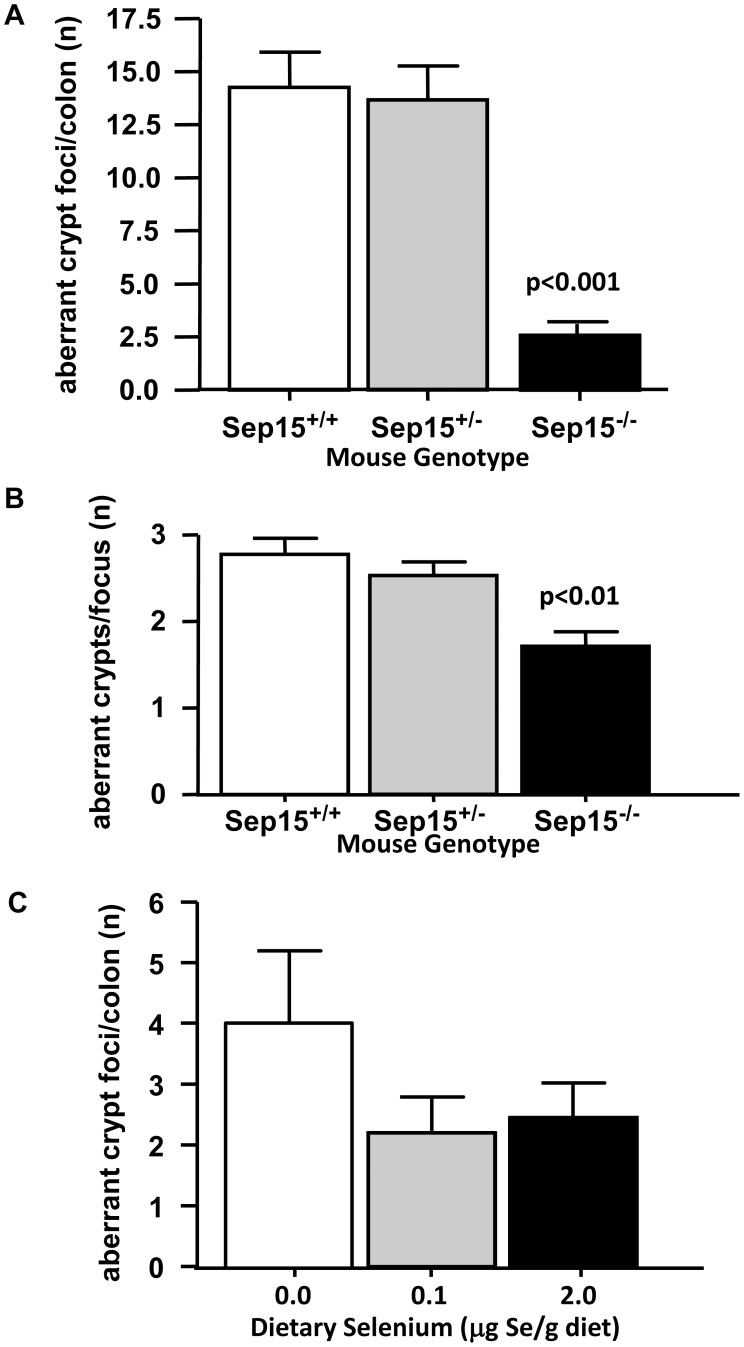
Aberrant crypt foci formation in AOM-treated wild type allelic (Sep15^+/+^), heterozygous (Sep15^+/−^) and Sep15 knockout (Sep15^−/−^) mice. (A) Number of ACF per colon in mice fed 0.1 µg selenium/g diet (mean±SEM; N = 12 per genotype); (B) number of aberrant crypts per focus in mice fed 0.1 µg selenium/g diet (mean±SEM; N = 12 per genotype); (C) numbers of ACF in Sep15 knockout mice on 0, 0.1 or 2.0 µg selenium/g diet (mean±SEM, N = 8–10).

The total number of aberrant crypt foci per colon was dramatically reduced in AOM-treated Sep15^−/−^ mice (2.58±0.50; p<0.001) compared to AOM-treated Sep15^+/−^ or Sep15^+/+^ littermate controls (14.25±1.67 and 13.67±1.60, respectively (Fig. 1A)). The average number of aberrant crypts per focus was also significantly lower in Sep15^−/−^ mice (1.71±0.18; mean±SEM; p<0.001) compared to Sep15^+/+^ mice (2.78±0.18; mean±SEM; Fig. 1B).

Dietary selenium did not significantly affect weight gain in Sep15 knockout mice over the course of the study, though growth appeared better in mice maintained on diets with levels of selenium near or above the recommended dietary allowance for rodents (0.15 µg selenium/g diet) [22]. This is reflected in the final weights at harvest for Sep15 knockout mice maintained on 0, 0.1 or 2.0 µg selenium/g diet, which were 25.8±0.36 g, 27.3±0.43 g and 26.6±0.57 g (mean±SEM, N = 8–10), respectively. A small but not statistically significant increase in the number of ACF was observed in AOM-treated Sep15 knockout mice fed a selenium-deficient diet (4.00±1.20) compared to adequate (2.20±0.59) or supranutritionally supplemented (2.45±0.56) selenium diets (Fig. 1C). Thus, Sep15 knockout mice were protected against chemically-induced aberrant crypt formation, which appeared to be largely independent of dietary selenium levels.

### Selenoprotein Expression

**Figure 2:**
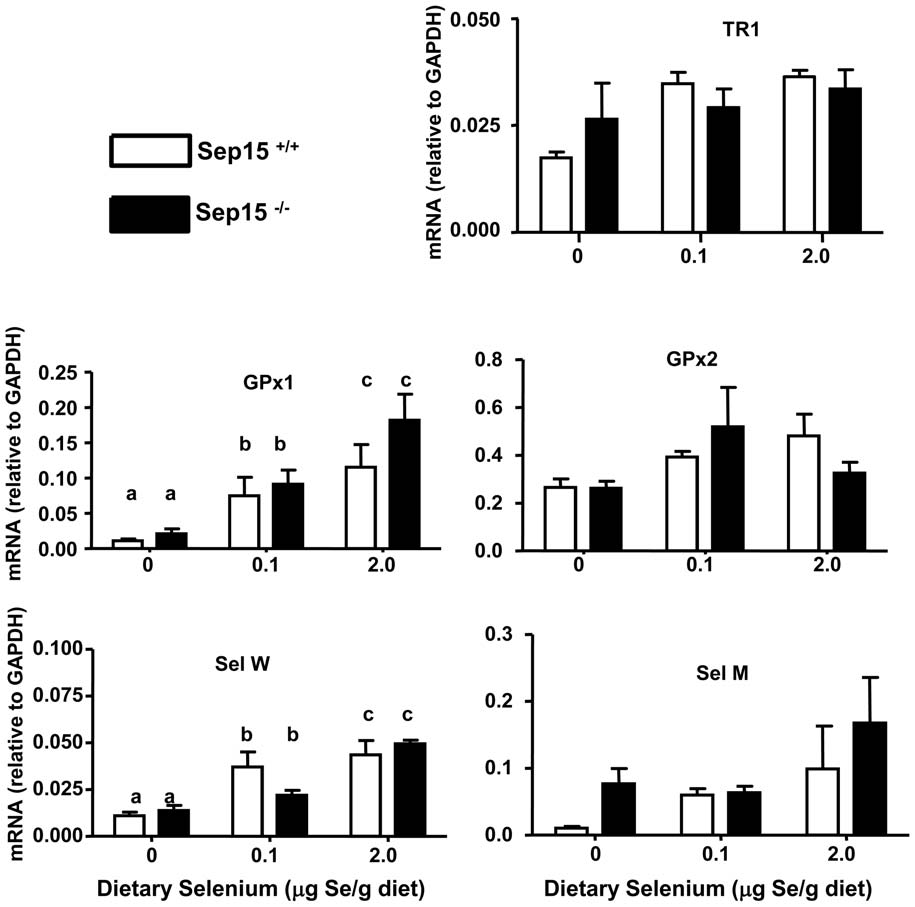
Analysis of selenoprotein mRNA expression in colonic epithelia of wild type allelic (Sep15^+/+^) and Sep15 knockout (Sep15^−/−^) mice. Mice were maintained on 0, 0.1 or 2.0 µg selenium/g diet and mRNA levels were measured using real-time RT-PCR. Values are means±SEM; N = 4 per genotype per diet. Letters indicate statistically significant differences (two-way ANOVA).

**Figure 3:**
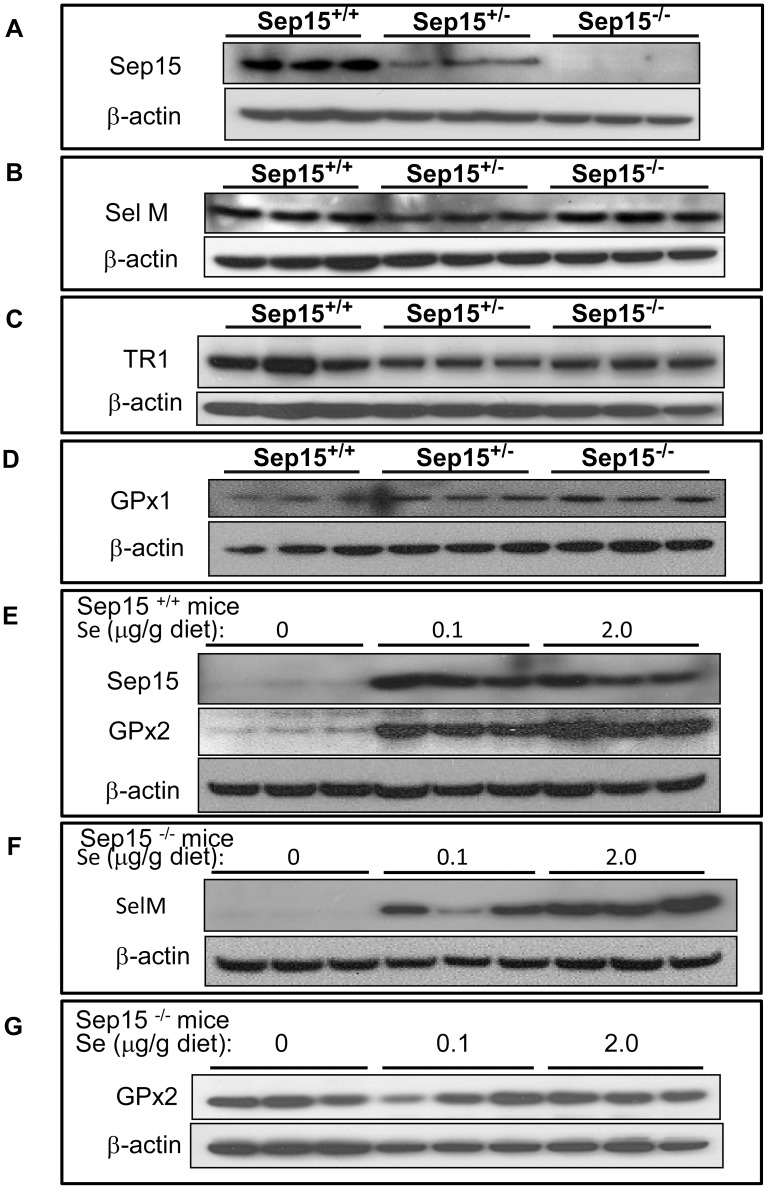
Protein expression of selenoproteins in colonic epithelia. Wild type allelic (Sep15^+/+^), heterozygous (Sep15^+/−^) and Sep15 knockout (Sep15^−/−^) mice were maintained on an 0.1 µg selenium/g diet. (A) Sep15 (80 µg protein/lane); (B) the Sep15 homolog SelM (40 µg protein/lane); and (C) TR1 (40 µg protein/lane); (D) GPx1 (40 µg protein/lane). Sep15^+/+^ and Sep15^−/−^ mice maintained on diets with 0, 0.1 or 2.0 µg selenium/g diet: (E) Sep15^+/+^ mice, Sep15 & GPx2 (75 µg protein/lane); (F) Sep15^−/−^ mice, SelM (75 µg protein/lane); (G) Sep15^−/−^ mice, GPx2 (75 µg protein/lane). β-actin was used as a loading control. All experiments were carried out in triplicate.

The effect of genotype and dietary selenium on the expression of various selenoproteins was assessed using real-time RT-PCR (Fig. 2) and Western blot analyses (Fig. 3). No statistical significant differences (two-way ANOVA, p>0.05) were observed in mRNA levels between Sep15^−/−^ mice and Sep15^+/+^ littermate controls for TR1, GPx1, GPx2, SelW, or SelM, a Sep15 homolog. A statistically significant diet effect with increasing dietary selenium was observed for GPx1 (ANOVA, p = 0.0002) and SelW (ANOVA, p<0.0001) for mice regardless of genotype. Sep15 mRNA expression is difficult to evaluate, as Sep15 knockout mice continue to synthesize a shortened albeit non-functional mRNA form [21]. Protein expression of Sep15 was detectable only in colonic epithelia of Sep15^+/+^ and Sep15^+/−^ mice (Fig. 3A) and absent in Sep15 knockout mice. Protein expression of other selenoproteins, such as the Sep15 homolog SelM (Fig. 3B), TR1 (Fig. 3C), and GPx1 (Fig. 3D), in mice maintained on adequate selenium diets did not differ among the three genotypes, as determined by Western blotting. Protein expression of Sep15 and GPx2 in Sep15^+/+^ mice (Fig. 3E), as well as SelM in Sep15^−/−^ mice (Fig. 3F), were nearly absent in animals on selenium-deficient diets. GPx2 expression in Sep15^−/−^ mice, on the other hand, seemed less susceptible to dietary selenium (Fig. 3G).

### Microarray Analyses

The expression of 281 genes were significantly different (ANOVA, p<0.001) between Sep15 knockout mice and wild type littermate controls as assessed by microarray analyses (Table S2). The gene whose expression changed the most was guanylate binding protein 1 (GBP-1), which was about 20-fold higher in colonic epithelia of Sep15 knockout mice compared to controls. Other GBP isoforms were not among the statistically significant genes (at p<0.001). The strongest down-regulated genes were the apolipoprotein A isoforms I (*Apoa1*) and IV (*Apoa4*). Other highly upregulated genes included polyamine-modulated factor 1 (*Pmf1*), a co-transcription partner of the Nuclear factor (erythroid-derived 2)-like 2 *(Nrf2*).

**Figure 4:**
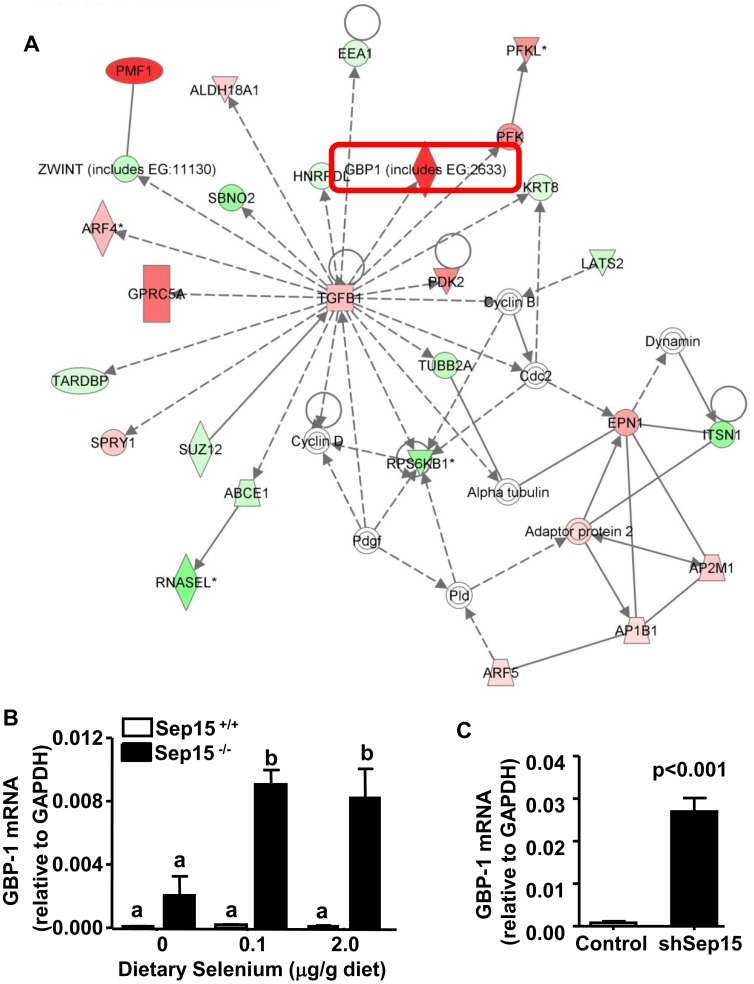
Ingenuity network analysis of genes regulated by knockout of Sep15 in mice and validation of GBP-1 mRNA expression in colonic epithelia of Sep15 knockout mice. (A) “Cellular development, growth and proliferation” was identified as the top affected network. Genes depicted in red have up-regulated expression and genes in green have down-regulated expression in Sep15 knockout animals compared to wild type littermate controls. Molecules that are not user specified, but are incorporated into the network through relationships with other molecules, are shown in white. Relative GBP-1 mRNA expression in (B) mouse colonic epithelia of Sep15^−/−^ and Sep15^+/+^ littermate control mice fed 0, 0.1 or 2.0 µg selenium/g diet (N = 8/genotype); and (C) in murine colon cancer CT-26 cells as determined by quantitative RT-PCR analysis. Means not sharing a common letter are statistically significant (p<0.0001).

Subsequent analysis using Ingenuity Pathway Analysis (IPA) demonstrated that the top five associated ontology networks were ‘Cellular development, growth, proliferation’ (see Fig. 4A), ‘Cellular assembly, cell cycle’, ‘Carbohydrate metabolism, cellular assembly’, ‘Cell cycle, cellular development’, and ‘Cancer, gene expression’. These networks were related to biological functions that included “developmental disorders”, “dermatological diseases” and “cancer.”

### GBP-1 Expression in Mouse Colonic Epithelia

**Figure 5:**
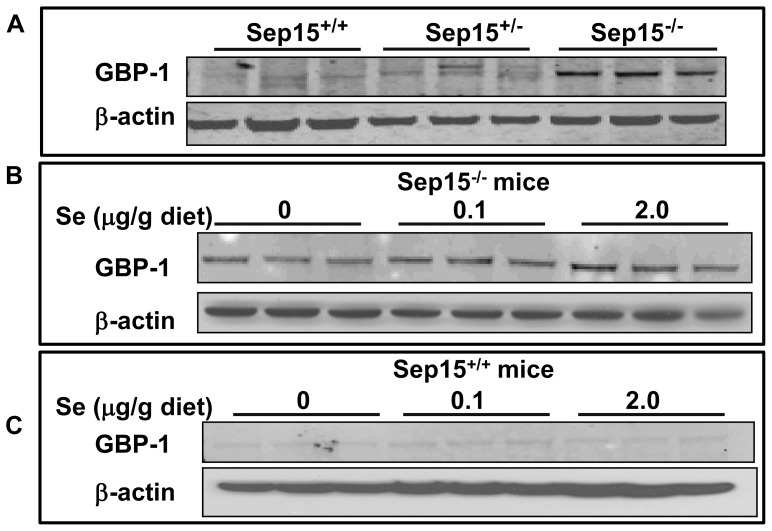
GBP-1 expression in mouse colonic epithelia. GBP-1 expression was examined by Western blots in colonic epithelia of (A) Sep15 knockout (Sep15^−/−^), heterozygous (Sep15^+/−^) and wild type allelic (Sep15^+/+^) littermate control mice on 0.1 µg selenium/g diet, (B) Sep15^−/−^ mice on 0, 0.1 or 2.0 µg selenium/g diet, and (C) Sep15^+/+^ litter mate controls on 0, 0.1 or 2.0 µg selenium/g diet. Protein extracts were loaded at 40 (A, B) or 60 µg (C) per lane. β-actin was used as the loading control. All experiments were carried out in triplicate.

Quantitative RT-PCR was utilized to validate the result of increased expression of the GBP-1 mRNA in the microarray analysis. Comparison of real-time RT-PCR and microarray data demonstrated a similar direction of the response in the GBP-1 mRNA expression, with Sep15^−/−^ mice demonstrating a 45-fold increased up-regulation (p<0.0001) compared to Sep15^+/+^ littermate controls (Fig. 4B), especially at adequate and supplemented dietary selenium levels. Additionally, quantitative RT-PCR was utilized to examine GBP-1 mRNA levels in our established murine CT-26 colon cancer cell line [20], in which Sep15 was down-regulated using RNAi technology. shSep15 cells demonstrated a greater than 30-fold increase of GPB-1 mRNA (p<0.001) compared to plasmid-transfected control cells (Fig. 4C). Subsequently, GBP-1 protein expression in colonic epithelia was assessed using Western blotting, which demonstrated that GBP-1 protein was strongly expressed in Sep15^−/−^ mice, but only very weakly expressed in Sep15^+/−^ or Sep15^+/+^ littermate controls (Fig. 5A). Although selenium deficiency seemed to demonstrate a reduction in GBP-1 mRNA expression (Fig. 4B), dietary selenium did not have an effect on the protein expression of GBP-1 in Sep15^−/−^ mice (Fig. 5B) or Sep15^+/+^ littermate controls (Fig. 5C).

### Cytokine Analysis

**Figure 6:**
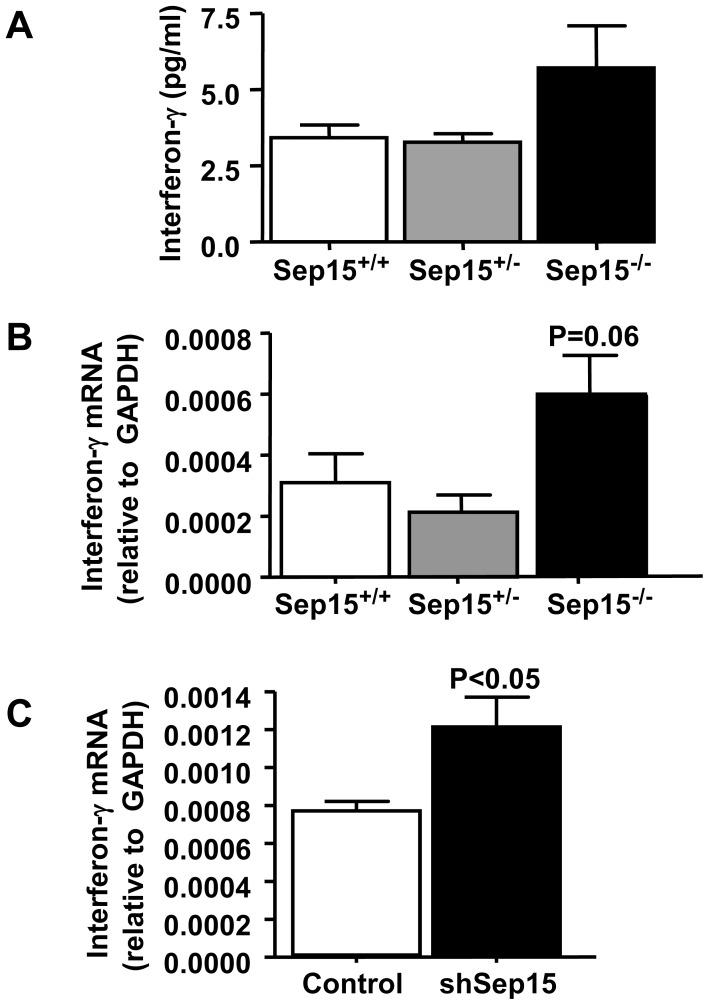
Interferon-γ levels. (A) Interferon-γ levels in serum of Sep15 knockout mice (Sep15^−/−^) compared to heterozygous (Sep15^+/−^) and wild type (Sep15^+/+^) litter mate controls (N = 5 per genotype) on 0.1 µg selenium/g diet as measured by ELISA. (B) Interferon-γ levels in colonic epithelia of Sep15^−/−^ mice compared to Sep15^+/−^ and Sep15^+/+^ litter mate controls (N = 5 per genotype) on 0.1 µg selenium/g diet as measured by quantitative real-time PCR. (C) Interferon- γ mRNA levels in murine colon cancer CT26 cells with targeted down-regulation of Sep15 (shSep15) compared to plasmid-transfected control cells as measured by real time RT-PCR.

Protein levels of interferon-γ, interleukin-1β, interleukin-2, interleukin-4, interleukin-5, KC/GRO, interleukin-10, interleukin-12p70 and tumor necrosis factor-α were assessed in a sandwich immunoassay format using mouse plasma samples from Sep15^−/−^, Sep15^+/−^ and Sep15^+/+^ littermate control mice on adequate selenium diets. Although not statistically significant, interferon-γ levels were elevated in plasma of Sep15^−/−^ mice (5.71±1.38 pg/ml) compared to Sep15^+/−^ (3.27±0.28 pg/ml) and Sep15^+/+^ (3.42±0.42 pg/ml) littermate controls (Fig. 6A). Similarly, interferon-γ mRNA levels appeared to be elevated in colonic epithelium of Sep15 knockout mice, compared to littermate controls (Fig. 6B). Interestingly, albeit only detectable at very low levels using real-time RT-PCR, a statistically significant increase in interferon-γ mRNA levels was observed in shSep15 CT26 murine colon cancer cells (p<0.05) compared to plasmid-transfected controls (Fig. 6C).

### β-catenin Expression in Mouse Colonic Epithelia

**Figure 7:**
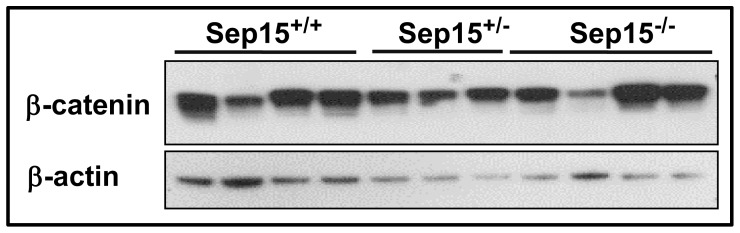
β-catenin protein expression in colonic epithelia of wild type (Sep15^+/+^) (N = 4), heterozygous (Sep15^+/−^) (N = 3) and Sep15 knockout (Sep15^−/−^) mice (N = 4) on 0.1 µg selenium/g diet. Cell lysates from colonic epithelia were loaded at 48 µg per lane. β-actin was used as the loading control.

Protein expression of β-catenin in colon epithelia of mice that had been maintained on a selenium-adequate diet was assessed using Western blotting. Although somewhat variable among individual mice, the overall β-catenin expression was similar in colon epithelial cell lysates of Sep15^−/−^, Sep15^+/−^ and Sep15^+/+^ mice (Fig. 7).

## Discussion

Our previous study demonstrated that targeted down-regulation of Sep15 in a murine colon cancer cell line resulted in the reversal of many characteristics typical of malignant cells [20]. It indicated that Sep15 might play an important role in the early steps of colon tumorigenesis. This stands in stark contrast to observations in lung cancer, that indicated lowered Sep15 expression to correlate with higher cancer incidence [23], and, while exactly why this discrepancy exists is subject to further study, it points to a possibly strong tissue-specificity in terms of the role of Sep15. The aim of the present study was to further investigate the role of Sep15 in colon cancer by assessing the role of Sep15 loss on carcinogen-induced ACF formation *in vivo*. The total number of ACF per colon and the number of aberrant crypts per focus were significantly lower in Sep15 knockout (Sep15^−/−^) mice compared to wild type (Sep15^+/+^) and heterozygous (Sep15^+/−^) littermate controls. As ACF serve as a surrogate biomarker for colon cancer risk in humans [24], these results indicate that a lack of Sep15 expression may be protective. Increased numbers of ACF have been reported in rats [6], [8] and mice [9] on selenium-deficient diets, but these conditions decrease expression of many selenoproteins. In our study, because the number of ACF was very low in Sep15 knockout mice overall, neither dietary selenium supplementation above adequate levels nor its deficit significantly affected the number of ACF. A larger number of animals or a prolonged duration of the study might have resulted in more distinct differences among the groups. Comparisons of supplemental dietary selenium including wild type/normal mice and rats are readily available in already published articles (*e.g*., [6]–[9]), and thus were not included in our experimental set up. Given that mice heterozygous for Sep15 did not show a reduction in aberrant crypt foci formation compared to wild type mice, we suspect that these heterozygous mice would behave similarly to wild type mice in response to dietary selenium levels. Nevertheless, a small increase in the number of ACF was observed in Sep15 knockout mice fed a selenium-deficient diet, suggesting the importance of dietary selenium in cancer prevention, as has been described previously [6], [9].

To investigate the possible effects of Sep15 loss on other selenoproteins *in vivo*, we measured the expression of several other selenoproteins in colonic epithelia. mRNA transcripts of TR1, GPx1, GPx2, SelW and SelM, a Sep15 homolog, did not respond with significant changes to deletion of Sep15 *in vivo* as measured by real-time RT-PCR. Statistically significant responses to dietary selenium on the mRNA and/or protein level were observed for GPx1, SelM, GPx2 and SelW, which is also consistent with previous observations [25], [26]. Protein expression of TR1 and SelM were similar among the three genotypes, indicating that the differences in the response to the chemical colon carcinogen azoxymethane were not because of differential expression of other selenoproteins.

We utilized microarray technology to determine the genes whose expression was modified by complete loss of Sep15. Interestingly, *Gbp1* was the highest up-regulated gene in Sep15 knockout animals compared to littermate controls, and this finding was validated by quantitative RT-PCR. On the other side of the spectrum, Apolipoprotein A-I and A-IV (*Apoa1* and *Apoa4*) were the most down-regulated genes in Sep15 knockout mice compared to controls.

Western analyses confirmed an up-regulation of GBP-1 on the protein level in Sep15^−/−^ mice compared to Sep15^+/−^ and Sep15^+/+^ controls. Even at a higher protein loading, GBP-1 is barely detectable in wild type mice, and does not respond to increased or decreased dietary selenium. These results were strengthened by a follow up study with our established shSep15 murine colon cancer cell line, which demonstrated that GBP-1 mRNA is also inducible through targeted down-regulation of Sep15 *in vitro.* Furthermore, in the NCI-60, a collection of 60 human cancer cell lines routinely used for screening and comparative analyses, the Sep15 gene is significantly correlated with GBP-1 (Pearson’s correlation, p<0.05), and also with its isoforms GBP-2, GBP-3 and GBP-4 (http://discover.nci.nih.gov/cellminer± database version 0.9). Thus, a significant link between Sep15 and GBP-1 and possibly other GBP-isoforms may be important in human cancers, possibly beyond colonic epithelia.

The *GBP-1* gene encodes guanylate binding protein 1, a large GTPase thought to function as an anti-apoptotic protein [27] and may contribute to anti-tumorigenic activities [28]. It is of interest to note that *Sep15* and *Gbp-1* are both located on chromosome 3 (3 H3 and 3 H1, respectively) in mice, but it is unlikely that these two genes have any interplay at the chromosomal level as they are separated by almost two million nucleotides. GBP-1 is considered to be an activation marker of endothelial cells during inflammatory diseases, and its function includes the inhibition of spreading and migration of endothelial cells through induction of integrin expression, a known key process during angiogenesis.

Interestingly, up-regulation of GBP-1 in humans also has been associated with a highly significant (p<0.001) increase in five-year survival rate in colorectal cancer patients [29]. Because guanylate-binding proteins are among the most abundant cellular proteins expressed in response to interferon-γ and other cytokines [30], [31], we analyzed the expression of various cytokines in plasma of Sep15 knockout mice compared to controls. Even though only a less than two-fold higher protein expression of interferon-γ was observed in plasma of Sep15 knockout mice, the result was intriguing in light of the fact that these mice were not treated with azoxymethane or any other stressors, and had been fed a diet with adequate selenium levels. This finding is further strengthened by the fact that we were also able to observe slightly elevated mRNA levels of interferon-γ in colonic epithelia of Sep15 knockout mice.

It is possible that Sep15 knockout mice exhibit elevated interferon-γ levels, thus resulting in a persistently increased GBP-1 mRNA and protein expression. Naschberger *et al*. have found that an interferon-γ-dominated reaction may counteract tumor progression in colorectal cancer [29]. Because our study investigated ACF as putative pre-neoplastic lesions, our results show that the absence of Sep15 and subsequent up-regulation of GBP-1coincided with a decreased ability to develop aberrant crypts upon chemical insult with a colon carcinogen. A recent study [32] demonstrated that GBP-1 is a potent suppressor of β-catenin levels and restricts epithelial cell proliferation in the intestine. Cytoplasmic β-catenin protein levels appeared largely unaffected in our study. However, activated (translocated) nuclear β-catenin levels may differ, as has been reported by Capaldo *et al*. [32]. Our observed strong increase in GBP-1 expression in Sep15-deficient animals would lend support to the argument that the intestinal epithelia in knockout animals were resistant to cell proliferation which would be necessary for ACF formation. Furthermore, according to the microarray analysis, the mRNA expression of *Apoa1* and *Apoa4* was strongly reduced in Sep15 knockout animals. *Apoa1* is increased in progressive stages of colonic adenocarcinoma with higher expression in carcinomas than in normal mucosal epithelial cells [33]. *Apoa4* is a gene coding for an anti-inflammatory protein in intestinal epithelium [34], and its expression has been linked with a more differentiated cancer phenotype. These apolipoproteins are also affecting both α- and β-catenins, integral components in the Wnt signaling pathway [35]. Kipp *et al.* recently established that the expression of the selenoproteins GPx2, TR2 and TR3 are regulated by Wnt signaling [36]. In our study, GPx2 levels were not different between Sep15 knockout mice and wild type controls. Thus, any dysregulation in the Wnt signaling pathway may have resulted because of lack of Sep15, not because of changes to GPx2. The decreased expression of both apolipoproteins paralleling the strong increase of GBP-1 in colonic epithelium suggests that loss of Sep15 may also be influenced by the Wnt signaling pathway thus resulting in downregulation of oncogenes and thus a protection against chemically-induced tumor initiation.

We intend to further investigate whether the link between GBP-1 and Sep15 indeed functions via interferon-γ directly through the apolipoprotein-catenin-Wnt-pathway regulation or other inflammation-associated pathways. To elucidate this possible functional link, we are also using human and mouse colon cancer cell lines to investigate whether any, and which, regulatory elements in the promoter region of GBP-1 may be affected by Sep15 expression. Other cytokines, such as interleukin-1β and tumor necrosis factor-α, are also thought to induce GPB-1 expression, albeit through a pathway that includes an interferon-α-stimulated response element and a NF-κB-binding motif [37]. Plasma expression of interleukin-1β and tumor necrosis factor-α did not appear to be different in Sep15 knockout mice compared to control mice.

In conclusion, the current investigation conclusively demonstrated that loss of Sep15 protects against formation of aberrant crypt foci in colonic epithelia *in vivo*. Consistent with these observations, an increased mRNA and protein expression of GBP-1 and decreased mRNA levels of *Apoa1* and *Apoa4* were observed in Sep15 knockout animals. Thus, Sep 15 appears to play a stimulatory role (possible oncogene) in cancer etiology in colonic tissue, whereas the possible links to GBP-1 and the apolipoproteins A-I and IV remain to be elucidated further. Given the recent findings in the SELECT human clinical trial compared to the earlier NPC trial [38], where a reduction in colon cancer risk was observed, it may be beneficial for future studies, if colon biopsies are possible, to include investigations into examining the levels of participants’ Sep15 and GPB-1 expressions, as this may affect their cancer risk, especially for colorectal cancer.

## Supporting Information

Table S1 **Primers used for measuring the mRNA levels for selenoproteins and genes in the Sep15 knockout mouse model by real-time PCR.**
(DOC)

Table S2 **List of statistically significantly greater than two-fold upregulated genes (ANOVA; p<0.001) in colonic epithelia of Sep15 knockout mice compared to wild type littermate controls (N = 4).** Listed are HUGO gene symbols, expected gene product and fold-change beginning with the highest upregulated genes in Sep15 knockout mice.
(DOC)
