# Supplementary material for: Knockout of the 15 kDa Selenoprotein Protects against Chemically-Induced Aberrant Crypt Formation in Mice
Source: PLoS One. 2012 Dec 4;7(12):e50574. doi: 10.1371/journal.pone.0050574 (PMC3514276; doi:10.1371/journal.pone.0050574)
Supplement: Table S1 — Primers used for measuring the mRNA levels for selenoproteins and genes in the Sep15 knockout mouse model by real-time PCR. (DOC) [file pone.0050574.s001.doc]

**Table S1:** Primers used for measuring the mRNA levels for selenoproteins and genes in the Sep15 knockout mouse model by real-time PCR

Target gene Accession No. Sequence

_____________________________________________________________

GAPDH NM_008084 fwd ATGTGTCCGTCGTGGATCT

rev GTTGAAGTCGCAGGAGACAA

GBP-1 NM_010259 fwd GATTTCTCCCTGGATCTGGA

rev CACAGGCGAGGCATATTAAA

GPx1 NM_008160 fwd CAGGAGAATGGCAAGAATGA

rev GAAGGTAAAGAGCGGGTGAG

GPx2 NM_030677 fwd ATCAAACGGCTCCTCAAAGT

Rev GGGACGATATTCAGGGAATG

IFNγ NM_008337 fwd GGCATAGATGTGGAAGAAAA

rev GGCCTGATTGTCTTTCAA

Sel M NM_053267 fwd GATTGGAACCGTCTTCGAG

rev GTGCTTCATCACCAGGTTGT

Sel W NM_009156 fwd TAGAGGCAGGGTCCTGAAAG

rev AATCCATCTCTGGCCTGACT

Sep15 NM_053102 fwd TGGAACACAGACAGTGTGGA

rev TGACCAATGTAAGCATGCAA

TR1 NM_015762 fwd CTACAGACCATTGCCTTGCT

rev ACCTCCTACCCACAAGATCC
