# Supplementary material for: Knockout of the 15 kDa Selenoprotein Protects against Chemically-Induced Aberrant Crypt Formation in Mice
Source: PLoS One. 2012 Dec 4;7(12):e50574. doi: 10.1371/journal.pone.0050574 (PMC3514276; doi:10.1371/journal.pone.0050574)
Supplement: Table S2 — List of statistically significantly greater than two-fold upregulated genes (ANOVA; p<0.001) in colonic epithelia of Sep15 knockout mice compared to wild type littermate controls (N = 4). Listed are HUGO gene symbols, expected gene product and fold-change beginning with the highest upregulated genes in Sep15 knockout mice. (DOC) [file pone.0050574.s002.doc]

**Table S2:** List of statistically significantly greater than two-fold upregulated genes (ANOVA; p<0.001) in colonic epithelia of Sep15 knockout mice compared to wild type littermate controls (N=4). Listed are HUGO gene symbols, expected gene product and fold-change beginning with the highest upregulated genes in Sep15 knockout mice.

| **Gene** | **Gene product** | **Fold change** |
| --- | --- | --- |
| *Gbp1* | guanylate binding protein 1 | 19.45 |
| *EG620313* | PREDICTED: Mus musculus predicted gene, EG620313 | 12.00 |
| *Pmf1* | polyamine-modulated factor 1 | 7.21 |
| *H28* | histocompatibility 28 | 6.88 |
| *Ubqlnl* | Ubiquilin-like (cDNA clone MGC:132965 IMAGE:40062172) | 5.05 |
| *Cyb5rl* | Cytochrome b5 reductase-like (cDNA clone IMAGE:3584113) | 4.52 |
| *Arid1a* | AT rich interactive domain 1A (SWI-like) | 4.01 |
| *Gprc5a* | G protein-coupled receptor, family C, group 5, member A (cDNA clone) | 3.94 |
| *Cfl1* | cofilin 1, non-muscle | 3.71 |
| *Sepw1* | selenoprotein W, muscle 1 | 3.44 |
| *Psme3* | proteaseome (prosome, macropain) 28 subunit, 3 | 3.30 |
| *Wibg* | Within bgcn homolog (Drosophila) | 3.30 |
| *Rcn1* | Reticulocalbin 1 | 3.26 |
| *Rasd2* | RASD family, member 2 | 3.21 |
| *Pdk2* | pyruvate dehydrogenase kinase, isoenzyme 2 | 3.08 |
| *Wdr53* | WD repeat domain 53 (cDNA clone MGC:25430 IMAGE:3987279) | 2.98 |
| *Atp6v0b* | ATPase, H+ transporting, lysosomal V0 subunit B | 2.96 |
| *Mospd3* | motile sperm domain containing 3 | 2.90 |
| *Lfng* | LFNG O-fucosylpeptide 3-beta-N-acetylglucosaminyltransferase | 2.88 |
| *Tinagl1* | tubulointerstitial nephritis antigen-like 1 | 2.87 |
| *Ass1* | argininosuccinate synthetase 1 | 2.85 |
| *Ncdn* | neurochondrin | 2.83 |
| *Ssbp3* | single-stranded DNA binding protein 3, transcript variant 2 | 2.74 |
| *Gjb1* | Gap junction protein, beta 1 (cDNA clone MGC:6217 IMAGE:3483365) | 2.71 |
| *Trp53i11* | transformation related protein 53 inducible protein 11 | 2.65 |
| *Tomm6* | translocase of outer mito. membrane 6 homolog, nuclear gene encoding mito.protein | 2.64 |
| *Tpcn1* | two pore channel 1 | 2.62 |
| *Pfkl* | phosphofructokinase, liver, B-type | 2.58 |
| *Ppif* | peptidylprolyl isomerase F (cyclophilin F), nuclear gene encoding mitochondrial protein | 2.56 |
| *Lsm2* | LSM2 homolog, U6 small nuclear RNA assoc. (S. cerevisiae) (cDNA cl MGC:13889 ) | 2.52 |
| *Obfc2b* | oligonucleotide/oligosaccharide-binding fold containing 2B | 2.49 |
| *Ssh3* | slingshot homolog 3 (Drosophila) | 2.42 |
| *Ampd2* | Adenosine monophosphate deaminase 2 (isoform L) | 2.39 |
| *Epn1* | epsin 1 | 2.38 |
| *Ucp2* | Uncoupling protein 2 (mitochondrial, proton carrier) (cDNA clone MGC:13955) | 2.35 |
| *Capn9* | calpain 9 | 2.31 |
| *Banp* | BTG3 associated nuclear protein, transcript variant 1 | 2.30 |
| *Creb3l1* | cAMP responsive element binding protein 3-like 1 | 2.26 |
| *Ppp2r1a* | protein phosphatase 2, regulatory subunit A (PR 65), alpha isoform | 2.24 |
| *Raver1* | ribonucleoprotein, PTB-binding 1 | 2.22 |
| *Aes* | amino-terminal enhancer of split | 2.19 |
| *Tbc1d13* | TBC1 domain family, member 13 | 2.18 |
| *Sema6a* | sema domain, transmembrane domain, and cytoplasmic domain, (semaphorin) 6A | 2.18 |
| *Ncoa3* | Nuclear receptor coactivator 3 | 2.17 |
| *Ifitm2* | interferon induced transmembrane protein 2 | 2.16 |
| *P2ry6* | pyrimidinergic receptor P2Y, G-protein coupled, 6 | 2.15 |
| *Ascc1* | activating signal cointegrator 1 complex subunit 1 | 2.14 |
| *Ogg1* | 8-oxoguanine DNA-glycosylase 1, nuclear gene encoding mitochondrial protein | 2.12 |
| *Gstm7* | glutathione S-transferase, mu 7 | 2.11 |
| *Prkaca* | Protein kinase, cAMP dependent, catalytic, alpha (cDNA clone MGC:6169) | 2.06 |
| *Rad23b* | RAD23b homolog (S. cerevisiae) (cDNA clone MGC:29174 IMAGE:4459657) | 2.03 |
| *Eri3* | exoribonuclease 3 | 2.02 |
| *Mlf2* | Myeloid leukemia factor 2 | 2.01 |
| *Taok2* | TAO kinase 2 (cDNA clone IMAGE:30292251) | 2.01 |
